# PAHs and Cognitive Impairment: Prenatal Exposure Catches Up with Toddlers

**Published:** 2006-08

**Authors:** Tanya Tillett

Previous studies have documented reduced fetal growth and developmental impairment resulting from exposure to environmental toxicants such as tobacco smoke. Now researchers at the Columbia Center for Children’s Environmental Health implicate another pre-natal exposure in causing health effects, demonstrating for the first time that exposure to airborne polycyclic aromatic hydrocarbons (PAHs) *in utero* may affect cognitive development during childhood **[*EHP* 114:1287–1292; Perera et al.]**.

PAHs are introduced into the environment by combustion––car, truck, or bus exhaust, power generation, and cigarette smoking are just a few sources—and are transferred across the placenta. Urban populations have greater exposure to PAHs and therefore may be especially at risk for subsequent adverse health and developmental effects.

As part of the broader multiyear Mothers and Children Study, the researchers studied a cohort of 183 children of nonsmoking women living in the Washington Heights, Central Harlem, and South Bronx neighborhoods of New York City. They obtained demographic, residential, health, and environmental exposure information by administering a questionnaire during the mothers’ last trimester of pregnancy. They also monitored the mothers’ personal air exposures during the third trimester using backpack monitors.

Umbilical cord blood was collected and analyzed for cotinine, heavy metal, and pesticide content. Lead concentration was analyzed in a subset of 135 subjects. During postnatal follow-up interviews, the research team recorded any changes in residence, tobacco smoke exposure, or other conditions. The children’s cognitive and psychomotor development was assessed at 1, 2, and 3 years of age using the Bayley Scales of Infant Development–Revised; the mothers also answered questionnaires on their children’s behavior.

Although they noted no significant effect on behavior or cognitive or psychomotor development at ages 1 or 2, the Columbia investigators found that the 3-year-olds who had higher prenatal exposure to PAHs scored on average 5.69 points lower on cognitive tests than the less-exposed children, even when controlling for other exposures and socioeconomic factors. The higher-exposed children also had twice the odds of developmental delay, suggesting an increased risk for performance deficits in language, reading, and math in the first years of school.

The authors acknowledge some limitations of the study, including small sample size, lack of air monitoring data for all three trimesters, and lack of postnatal data for personal air PAH concentrations and lead exposure. They conclude that additional studies should be conducted to confirm their results, especially since limited performance in the early school years can provide an indication of future suboptimal school performance.

## Figures and Tables

**Figure:**
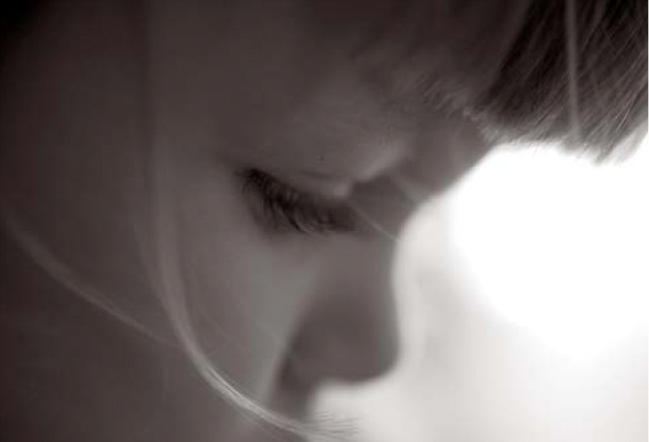
Thought leader Prenatal exposure to PAHs may affect cognitive development later on.

